# In-line filtration in very preterm neonates: a randomized controlled trial

**DOI:** 10.1038/s41598-020-61815-4

**Published:** 2020-03-19

**Authors:** Anne-Laure Virlouvet, Julien Pansiot, Artemis Toumazi, Marina Colella, Andreas Capewell, Emilie Guerriero, Thomas Storme, Stéphane Rioualen, Aurélie Bourmaud, Valérie Biran, Olivier Baud

**Affiliations:** 1 Assistance Publique-Hôpitaux de Paris, Neonatal intensive care unit, Robert Debré children’s hospital, Paris, France; 2 Delegation Paris 7, Inserm U1141, University of Paris, Paris, France; 3 Assistance Publique-Hôpitaux de Paris, Unit of Clinical Epidemiology, Robert Debré children’s hospital, University of Paris, Inserm U1123 and CIC-EC, 1426 Paris, France; 4 Pall Medical, SLS, Dreieich, Germany; 5 Assistance Publique-Hôpitaux de Paris, Department of Pharmacy, Robert Debré children’s hospital, Paris, France; 6 Department of Neonatal Medicine, Brest University Hospital, Brest, France; 7 Division of Neonatology and Pediatric Intensive Care, Children’s University Hospital of Geneva and University of Geneva, Geneva, Switzerland

**Keywords:** Paediatric research, Diagnostic markers, Prognostic markers, Risk factors, Inflammation

## Abstract

In-line filtration is increasingly used in critically-ill infants but its benefits, by preventing micro-particle infusion in very preterm neonates, remain to be demonstrated. We conducted a randomized controlled trial among very preterm infants allocated to receive either in-line filtration of all the intra-venous lines or standard care without filters. The primary outcome was differences greater than 20% in the median changes in pro-inflammatory cytokine serum concentrations measured at day 3 and day 8 (+/−1) using a Luminex multianalytic profiling technique. Major neonatal complications were analyzed as secondary predefined outcomes. We randomized 146 infants, assigned to filter (n = 73) or control (n = 73) group. Difference over 20% in pro-inflammatory cytokine concentration between day 3 and day 8 was not found statistically different between the two groups, both in intent-to-treat (with imputation) and per protocol (without imputation) analyses. The incidences of most of neonatal complications were found to be similar. Hence, this trial did not evidence a beneficial effect of in-line filtration in very preterm infants on the inflammatory response syndrome and neonatal morbidities. These data should be interpreted according to local standards in infusion preparation and central line management.

## Introduction

Despite improvements in perinatal care during the past three decades, prematurity is still associated with substantial mortality and morbidities for which inflammation plays a causal role^1,2^, including brain damage^3–6^, bronchopulmonary dysplasia (BPD)^7–9^ and retinopathy of prematurity (ROP)^10–14^. Perinatal inflammation is not only a major risk factor for prematurity but also the best predictor of poor neurological outcome, leading to permanent sequelae in 9 million infants every year^2,4,15–17^. Therefore, reducing factors involved in systemic inflammation appears to be a relevant strategy to improve outcomes of infants delivered very preterm.

Intra-venous (IV) drugs and parenteral nutrition infusion through central lines are among the most essential interventions in preterm neonates but they are associated with potential risks including bloodstream infections, thrombi and infusion of macro/micro particulates^18^. Particles in the infusion, containing metals, drug crystals^19^, glass fragments or cotton fibres^20^, can be generated by drug preparation process from packaging, incomplete reconstitution and chemical incompatibilities^21^. Parenteral infusion of micro-macro particles was reported to be associated with an increased risk of microvessels obstruction and inflammation, as reported in the lung and increased circulating cytokines release by the endothelium^22^. In-line filtration has therefore been proposed to limit the load of particles infused through central lines^23,24^, and to prevent bacterial and endotoxin infusion^25^. In-line filtration was found associated with a reduction of systemic inflammation and severe complications in critically-ill pediatric patients^26^. In neonates, a significant decrease in major complications and substantial cost savings was also reported^27^. However, there is still insufficient evidence to recommend the use of IV in-line filters in very preterm infants, especially in settings of neonatal intensive care units with high standards for infusion preparation and central line management^28^. Here, we hypothesized that a reduction in circulating cytokine levels in very preterm infants might be beneficial to prevent prematurity-related co-morbidities, including brain damage, BPD and ROP.

## Patients and Methods

### Study design and patients

The FRISBEE trial was a randomized controlled clinical trial conducted in a single tertiary level neonatal intensive care unit. We enrolled inborn infants delivered between 24^0/7^ and 31^6/7^ weeks of gestation or with a birthweight below 1500 g. Exclusion criteria were outborn birth, congenital malformation or known chromosomal aberrations and severe perinatal asphyxia.

The trial was approved by the national ethics committee (Comité de Protection des Personnes, Ile-de-France 1, Hôtel Dieu, Paris) and the French data protection authority (Commission Nationale de l’Informatique et des Libertés, N°1921016). Because this trial investigated routine care without additional blood samples, written informed consent was obtained from the guardians prior the study. This trial had been registered in ClinicalTrial.gov, NCT02686060, on 19/02/2016 before the first patient was enrolled. The completion and reporting of the FRISBEE trial is in accordance with CONSORT 2010 guidelines.

### Randomization and masking

The randomization sequence was electronically generated with nQuery (version 6.01). Enrollment was obtained by clinicians and group assignment was managed using a secure study website (Cleanweb, Telemedecine Technologies, Boulogne-Billancourt, France) after verification of eligibility and consent status. Infants were randomly assigned 1:1 to either the filter or control group via central computer-generated lists within the first hour of life. Filtration cannot be masked or replaced by sham but cytokine measurements were performed by an investigator unaware of the study groups.

### Procedures

Prior to this trial, the infusion scheme were optimized and standardized for all patients to avoid any drug incompatibilities, as previously reported^29^.

The filter group received in-line filtration of all the IV medications and individualized parenteral nutrition, with the exception of insulin, vitamin K, phenobarbital, blood and blood products. Appropriate filters used were 0.2 μm positively charged filters (Posidyne NEO96E, PALL Medical, Dreieich, Germany) for parenteral nutrition and other aqueous solutions, and 1.2 μm filters (Lipipor NLF1E Filters, PALL Medical, Dreieich, Germany) for infusion of lipid-containing mixtures. Filters were used for infusion through monolumen umbilical catheters, percutaneous central lines and peripheral venous lines. According to the available guidelines^18^, filters were placed as close as possible to the patient. The administration sets, including filters were changed after 72 hours of regular use for NEO96E and after 24 hours for NLF1E. Infants assigned to the control group were treated similarly but without filters and lines were changed at the same frequency.

### Biological samples and primary outcome

At day 0, 3, 8 (+/−1) and 30 (+/−3) of life, blood samples were collected, centrifuged and stored at −80 °C. Serum concentrations of a panel of 27 cytokines (Bioplex Pro Human Cytokine Grp1 Panel 27Plex, Bio-Rad, France) were measured using the Luminex multianalytic profiling technique. Serum was diluted at 1:4 and each sample was assessed twice. The detection and quantification of cytokine levels were performed using a Bio-Plex 200 system with a standard curve on each plate (Bio-Rad). Analysis of data was performed with bio-Plex Manager 6.0 software, by a physician blinded to the treatment group.

Differences greater than 20% in the median changes in pro-inflammatory cytokines serum concentrations (IL-1β, IL-6, IL-8 and TNFα) measured at day 3 and day 8 (+/−1) were compared between the two groups and used as the primary outcome. Indeed, this period is recognized as highly critical for very preterm infants exposed to pro-inflammatory events or procedures. The 20% change in pro-inflammatory cytokines serum concentrations has been decided after a consensus had been obtained from a panel of neonatologists and from the scientific committee of the trial asked about the minimal threshold they considered as clinically relevant and important. Indeed, the median levels of IL-1β, IL-8, and TNFα were previously reported to be about twice as high as those previously described for term infants^5^. Based on these data, we hypothesized that a reduction in cytokine level closer to the one measured in term newborn might be beneficial to prevent prematurity-related co-morbidities. The consensus about the minimal clinical relevant difference that we could expect from in-line filtration concluded that a 20% reduction is more reasonable compared to the 50% reduction suggested in the ELGANs study.

Serum concentration of other cytokines assessed as median changes between day 3 and day 8 (+/−1) were secondary biological outcomes. All cytokines were also measured at day 30 (+/−3) or when catheter was removed.

### Clinical outcomes

Clinical outcomes were analyzed as secondary predefined outcomes. They included neonatal mortality before 36 weeks of postmenstrual age (PMA), and 11 major neonatal morbidities including air leaks, pulmonary hemorrhage (bleeding into the lungs associated with respiratory distress syndrome), pulmonary hypertension requiring inhaled nitric oxide, BPD at 36 weeks of PMA (defined as a need for supplemental oxygen or ventilatory support according to Walsh *et al*.^30^), hemodynamically significant patent ductus arteriosus requiring either nonsteroid antiinflammatory drugs or surgical closure, insulin treatment, late-onset sepsis, necrotizing enterocolitis^31^ and isolated gastro-intestinal perforation, severe brain lesions (intraventricular hemorrhage grade 3–4 and cystic white matter damage) and ROP grade ≥ 2. Late-onset sepsis was confirmed when clinical signs were associated with positive standard blood culture and C-reactive protein >10 mg/L, leading to antibiotic treatment for >5 days.

### Filter analyses

Two *ex vivo* experiments using filters were performed and analyzed by electron microscopy and energy dispersion spectroscopy at PALL Medical SLS. The first one investigated filters used *ex vivo* but in conditions mimicking usual use in the NICU, combining medications and parenteral nutrition commonly infused during the first 4 days of life in a “standard” preterm infant of 1000 g. A second series of filters used during the clinical trial were collected after standard change and stored at 4 °C. Unused filters of each type were analyzed as controls. Upstream membranes were analyzed by an automated electron microscope (PSEM7512LS, LOD: 5 µm; Aspex LLC, Delmont, PA, USA) for particles >5 μm and a manual scanning electron microscope (Jeol JSM 840 A, Tokyo, Japan) combined to EDX-spectrometer (Oxford 6209, Abingdon-on-Thames, UK) for elemental compositions.

### Sample size

We hypothesized that in-line filtration could induce a significant reduction of the proportion of infants with at least 20% increase in median serum concentrations of at least one pro-inflammatory cytokine between day 3 and day 8 compared to the control group. The predetermined fixed sample size, controlling for the alpha-risk of 0.05 and a power of 80% and assuming a standard deviation of the difference equal to 1, was 63 patients per group. To account for dropouts, technical failures and the heterogeneity of patients, 73 infants in each group were recruited to ensure the intention-to-treat (ITT) final analysis.

### Statistical analysis

Baseline characteristics and complications were reported as frequencies (percentages) or medians and interquartile range (IQR) for the qualitative and quantitative variables respectively.

The primary endpoint analysis was conducted according to the principles of intention-to-treat (ITT) and “per-protocol”. All the other secondary analyses were done on “per protocol” basis.

For the primary endpoint analysis, in order to deal with missing values, the Markov Chain Monte Carlo multiple imputation method was used for inflammatory cytokine datasets at day 3 and day 8. Five multiple imputations were performed for each missing data, as recommended^32^. The multiple imputed datasets were analyzed using standard procedures for complete data analysis. They were used for the primary analysis only. For the secondary endpoint analysis, the original datasets, with missing data, were used. Comparisons were done using a Chi² or Fisher exact test for categorical data and with a non-parametric Wilcoxon-Mann-Whitney test or t-test for quantitative data. Post hoc sub-group analysis were performed, focusing on growth restriction <10e percentile and infants born before <28 weeks of gestation.

Since we had no a priori on the distributions of the cytokines level, a non-parametric approach was considered for all statistical analyses, allowing no wrong assumptions. All the statistical tests were two-tailed using a significance level of 5%. Statistical analyses were performed using SAS software. SAS and all other SAS Institute Inc. product or service names are registered trademarks or trademarks of SAS Institute Inc. in the USA and other countries.

### Clinical trial registry name

FRISBEE.

### Registration number

Clinicaltrial.gov identifier: NCT02686060.

### Data sharing statement

Deidentified individual participant data (including data dictionaries) will be made available, in addition to study protocols, the statistical analysis plan, and the informed consent form. The data will be made available upon publication to researchers who provide a methodologically sound proposal for use in achieving the goals of the approved proposal. Proposals should be submitted to olivier.baud@hcuge.ch.

## Results

Among the 146 infants enrolled between April 2016 and October 2017, 73 were assigned to the filter group and 73 to the control group (Supplementary Fig. 1). The primary outcome has been assessed in 72 and 73 infants, for intent-to-treat analysis, and in 62 and 58 infants for per protocol analysis, in the filter and control groups, respectively. The baseline characteristics of both mothers and infants were well balanced between the two groups (Table 1).

**Table 1:** Baseline characteristics of recruited infants and their mothers.

	Control group (N = 73)	Filter group (N = 72)	p-value
**Mothers**			
Multiple gestation	27 (37%)	25 (35%)	0.78
Gestational hypertension	19 (26%)	17 (24%)	0.74
Gestational diabetes	5 (7%)	2 (3%)	0.25
Antibiotics	54 (74%)	53 (74%)	0.96
Tocolysis	46 (64%)	47 (65%)	0.86
Prolonged rupture of membranes >24 h	24 (33%)	21 (29%)	0.63
Antenatal corticosteroids	67 (92%)	64 (89%)	0.56
Histological chorioamnionitis	13/62 (21%)	14/66 (21%)	0.97
Cesarean section	34 (47%)	29 (40%)	0.44
**Infants**			
Male	34 (47%)	31 (43%)	0.67
Gestationnal age (week)	30.0 (27.6–31.3)	30.2 (27.2–31.1)	0.80
Birthweight (g)	1250 (940–1372)	1110 (850–1368)	0.24
Intrauterine growth retardation			
<10^th^ perc.	19 (26%)	22 (31%)	0.74
<3^rd^ perc.	12 (16%)	15 (20%)	
Head circumference (cm)	26 (24–28)	26 (23–28)	0.34
Apgar score at 1 min	8 (5–9)	7 (4–9)	0.54
Apgar score at 5 min	9 (8–10)	9 (7.5–10)	0.64
CRIB II score	7 (4–10)	7 (4–10)	0.93
Blood cord pH value	7.33 (7.27–7.38)	7.32 (7.24–7.38)	0.46
Early onset sepsis	10/73 (14%)	11/70 (16%)	0.82

Data are expressed as n (%), n/N (%), or median (interquartile range).

CRIB denotes Clinical Risk Index for Babies.

**Figure 1:**
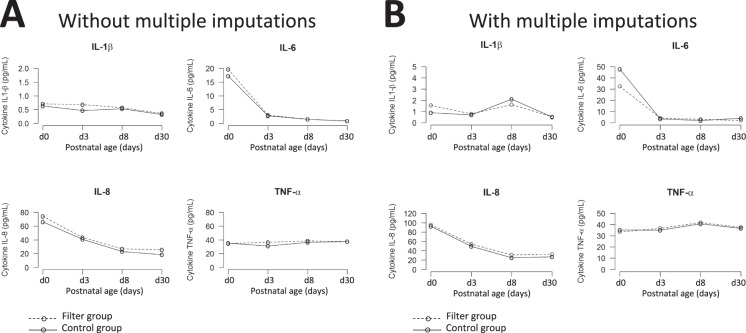
Time course of pro-inflammatory cytokine serum concentrations between birth and Day 30, in per protocol (**A**) and intend-to-treat (**B**) analyses.

The primary outcome was based on variations in serum concentrations of pro-inflammatory cytokines (IL-1β, IL-6, IL-8 and TNFα) between day 3 and day 8 (+/−1). This primary outcome was analyzed as intent-to-treat after multiple imputations (ITT) and as per protocol (PP). The Supplementary Table 1 shows no statistically significant differences in median variations of these 4 pro-inflammatory cytokines between the two groups. Figure 1 shows the time course from birth to day 30 (or catheter removal) for each pro-inflammatory cytokine concentration, with and without in-line filtering. These concentrations were not different between the two groups, both in ITT and PP analysis. Similarly, the distribution of infants with a more than 20% increase or decrease in pro-inflammatory cytokine concentrations between day 3 and day 8 (+/−1) were not different with and without in-line filtering (Table 2). Serum concentrations of the 24 other cytokines showed no significant difference in their median changes between day 3 and day 8 (+/−1) (Supplementary Table 2).

**Table 2:** Changes in cytokine serum concentration between Day 3 and Day 8 in imputed data set.

Cytokine	Control group (N = 73)	Filter group (N = 72)	p-value
**IL-1β**			
>20% increase >20% decrease	43 (58%) 21 (29%)	44 (61%) 22 (31%)	0.95
**IL-6**			
>20% increase >20% decrease	16 (22%) 48 (66%)	21 (29%) 42 (58%)	0.30
**IL-8**			
>20% increase >20% decrease	17 (23%) 46 (63%)	16 (22%) 45 (63%)	0.92
**TNFα**			
>20% increase >20% decrease	37 (51%) 22 (30%)	27 (38%) 23 (32%)	0.36

**Figure 2:**
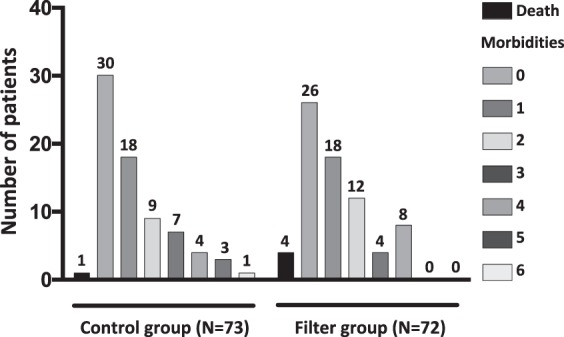
Distribution of patients according to their cumulative morbidities recorded before discharge.

Comparison of neonatal mortality and morbidities between infants with and without in-line filtering was shown in Table 3. Neonatal mortality between the two groups was not statistically different. The two groups did not differ significantly in the incidence of most neonatal morbidities. Nevertheless, in-line filtering was associated with a significantly lower incidence of pulmonary hemorrhage (0/72 *vs* 5/73, p = 0.02) and with a trend towards lower incidence of severe ROP (6/72 *vs* 12/73, p = 0.17). No difference between the two groups was observed regarding the distribution of patients according to their cumulative morbidities recorded before discharge (Fig. 2). In sub-group analysis focusing on growth restriction <10^th^ percentile and infants born before <28 weeks of gestation, no difference was observed in biological and clinical outcomes.

**Table 3:** Comparison of neonatal mortality and morbidities between groups. Data are expressed as n (%).

Variable	Control group (N = 73)	Filter group (N = 72)	p-value
Pneumothorax	0 (0%)	0 (0%)	—
Pulmonary hemorrhage	5 (6.8%)	0 (0%)	0.02
PPHN treated with inhaled Nitric Oxide	7 (9.6%)	5 (6.9%)	0.56
BDP at 36 weeks PMA	8 (11.1%)	10 (14.7%)	0.53
Medically-treated PDA	19 (26%)	16 (22.2%)	0.59
Surgery for PDA closure	2 (2.6%)	0 (0%)	0.16
Necrotizing enterocolitis grade >2a	4 (5.5%)	4 (5.6%)	0.98
Gastrointestinal perforation	0 (0%)	3 (4.1%)	0.08
Severe cerebral lesions	11 (15.1%)	8(11.1%)	0.48
Retinopathy of prematurity grade >2	12 (16.7%)	6 (8.8%)	0.17
Late-onset sepsis	30 (41.1%)	30 (41.7%)	0.94
Glucose intolerance requiring insulin infusion	18 (24.6%)	18 (25%)	0.96
Death before discharge	1 (1.4%)	4 (5.6%)	0.30

PPHN denotes persistent pulmonary hypertension.

BPD denotes broncho-pulmonary dysplasia.

PMA denotes postmenstrual age.

PDA denotes patent ductus arteriosus.

The rate of adverse events leading to unexpected removal of catheter or special care (luminal obstruction, extravascular fluid effusion, local cutaneous inflammation) was similar between groups (10 [14%] in each group). No obstruction related to filters was recorded.

We performed a microscopy analysis of filters depicted in Supplementary Fig. 2 and Table 3. For *ex vivo* experiments, composition of the particles >5 µm was a mixture of carbon, oxygen, copper, sulphur, selenium and chlorine. In addition to these particles, some elementals not part of the membrane itself were found in 8/12 NEO96E filters. For *in vivo* filters collected from patients, particles were counted on 2/5 NLF1E upstream filters and on 5/11 NEO96E filters. Most of the particles were found in filters used for parenteral or lipids infusion, and usually at very low density. Their composition included calcium, fluorine, iron and chromium. Elementals, which are not part of the membrane, were also found in 3/5 NLF1E filters and in 4/11 NEO96E filters. No silicon was found. No particle matters were observed in the 4 control filters. It should be noted that due to the automated counting method small particles <5 µm are below the level of detection (Supplementary Fig. 3).

## Discussion

In this randomized controlled clinical trial, in-line filtration of parenteral nutrition and other intra-venous drugs, compared to infusions without filters, was not found to be associated with significant change in the profile of pro-inflammatory cytokine levels measured between day 3 and day 8. Most clinical outcomes were similar between the two groups despite a reduction in the incidence of pulmonary hemorrhage in the filtered group.

These results should be interpreted regarding the optimal setting of infusions and drug preparation implemented in the neonatal intensive care unit before including patients in the trial. IV preparations were performed using the highest standards of quality with strict control of chemical stability, the use of automates in sterile environment, filtration of all components from glass vials, the immediate use after preparation and controlled training of nurses. Not surprisingly, we didn’t record any filter obstruction and only a low density of particles >5 µm were found in a limited number of filters.

We have chosen pro-inflammatory cytokine serum concentrations as primary outcome to assess the effect of in-line filtration on systemic inflammation. The rationale for investigating systemic inflammation during the first postnatal week is supported by several studies demonstrating its causal role in the neurodevelopmental vulnerability of very preterm infants. Systemic inflammation is recognized to activate brain microglia^33–38^, the resident macrophages of the central nervous system and to sensitize the developing brain to a secondary hypoxic or excitatory insult^39^ leading to neuro-inflammation, diffuse white and grey matter damage^40^. Previous studies also supported the use of biological markers of inflammation to assess the effect of in-line filtration in non-neonatal populations^20,22^. In previous studies, a reduction in systemic inflammation and a trend towards less renal and pulmonary complications, as well as in the occurrence of low platelet levels were shown in severely ill children when infusions were filtered^26,41,42^. The unique randomized clinical trial specifically enrolling neonates^27^ found a significant reduction in a composite score combining necrotizing enterocolitis, clinical sepsis, proven sepsis and thrombi, suggesting an effect in the systemic inflammatory response. The present trial did not confirm this association but standards of care and quality of preparations to prevent particle generation have likely changed over time. In addition, we observed a rapid decrease in pro-inflammatory cytokine concentrations spontaneously between birth and day 3 in both groups, reducing the added-value of in-line filtering to reduce systemic inflammation.

An association between circulating cytokine levels and specific clinically relevant morbidities has been observed in many previous studies. Regarding brain damage, intermittent or sustained systemic inflammation has been shown to contribute to brain damage in extremely preterm infants^3,4^. Elevated blood levels of inflammation-related proteins has been also shown to be associated with later brain volumes and cognition^5^ and associated with an attention problem at age 24 months in extremely preterm infants^6^. Increased risk of BPD was associated with elevated blood concentrations of a variety of proinflammatory cytokines^9^. Finally, neonatal exposure to inflammation appears to contribute to the increased ROP risk in very preterm infants^12,14^, especially within the first two postnatal weeks^13^.

Besides the biological effects of in-line filtration, we did not observe significant differences in the occurrence of the main neonatal complications. The incidence of pulmonary hemorrhage was found significantly reduced but conclusions must be drawn with caution due to the small sample size. In contrast to Van Lingen trial, we did not find any association between in-line filtration and a reduction in catheter-associated bloodstream infections. In the FRISBEE trial, changes of in-line sets were similar between the two groups (every 72 hours) when change occurred every 24 hours in control group and every 96 hours in filter group in the Van Lingen study, leading to more frequent manipulations and opening of the lines only in the control group. Our findings are consistent with the absence of bacterial contamination in 199 tested infusion bags leftover after their clinical use as reported by Oie *et al*.^21^. Catheter-associated sepsis in neonates have also been recognized to be more related to bacterial colonization of the cannula site or ports catheter tubing rather than from the direct luminal infusion^43^, limiting the interest of in-line filtration for this purpose.

Despite the negative results shown in this trial, we cannot rule out several benefits provided by filters. First, filters can avoid direct infusion of air bubbles or bacteria through central catheters, two rare but potentially serious adverse events. Drug incompatibilities and subsequent risk of in-line obstruction can also be prevented.

This controlled clinical trial is the largest in investigating in-line filtration in very low gestational/birthweight infants, in a setting of very high standards of care. Its main limitation is that it was underpowered to detect differences in rare clinical outcomes. Also, cytokine datasets used for the primary criteria analysis were impeded by a substantial proportion of missing values, replaced using a multiple imputation procedure by fully conditional specification. Nevertheless, both intent-to-treat (with imputation) and per protocol (without imputation) analyses had similar results.

In conclusion, this study did not evidence a beneficial effect of in-line filtration in very preterm infants on the inflammatory response syndrome and neonatal morbidities, a result potentially explained by optimizing practices before the start of the clinical trial. However, these data should be interpreted cautiously according to level of standards in infusion preparation and central line management.

## Supplementary information

Supplementary information.
