# Supplementary material for: In-line filtration in very preterm neonates: a randomized controlled trial
Source: Sci Rep. 2020 Mar 19;10:5003. doi: 10.1038/s41598-020-61815-4 (PMC7081338; doi:10.1038/s41598-020-61815-4)
Supplement: Supplementary file 1 — Supplementary information. [file 41598_2020_61815_MOESM1_ESM.docx]

**Supplementary materials**

**In-line filtration in very preterm neonates: a randomized controlled trial**

Anne-Laure Virlouvet^1,2^, MD, Julien Pansiot^2^, MSc, Artemis Toumazi^3^, MSc, Marina Colella^1,2^, MD-PhD, Andreas Capewell^4^, PhD Emilie Guerriero^5^, PharmD, Thomas Storme^5^, PharmD, MD, Stéphane Rioualen^6^, MD, Aurélie Bourmaud^3^, MD, Valérie Biran^1,2^, MD-PhD, Olivier Baud^1,2,7*^, MD-PhD.

^1^ Assistance Publique-Hôpitaux de Paris, Neonatal intensive care unit, Robert Debré children’s hospital, Paris, France ; ^2^ Delegation Paris 7, Inserm U1141, University of Paris, Paris, France ; ^3^ Assistance Publique-Hôpitaux de Paris, Unit of Clinical Epidemiology, Robert Debré children’s hospital, University of Paris, Inserm U1123 and CIC-EC 1426, Paris, France ; ^4^ Pall Medical, SLS, Dreieich, Germany ; ^5^ Assistance Publique-Hôpitaux de Paris, Department of Pharmacy, Robert Debré children’s hospital, Paris, France ; ^6^ Department of Neonatal Medicine, Brest University Hospital, Brest, France ; ^7^ Division of Neonatology and Pediatric Intensive Care, Children's University Hospital of Geneva and University of Geneva, Geneva, Switzerland.

**Supplementary Figures :**

**Supplementary Figure 1**: Flow chart of recruited patients.

**Supplementary Figure 2**: Set-up of *ex vivo* experiments.

**Supplementary Figure 3**: SEM images of particles in the uptream side of membranes from NEO96E filters. Arrows point particles > 5μm. Circles point micro-particles aggregates below level of detection.

**Supplementary Tables :**

**Supplementary Table 1**: Change in cytokine serum concentration between Day 3 and Day 8 with (ITT) and without (PP) multiple imputations of missing data.

**Supplementary Table 2**: Changes in the serum concentrations (pg/mL) of the panel of 27 cytokines in Filter group compared to Control group between Day 3 and Day 8. Values are expressed as median [interquartile range] and statistical comparisons were performed using Wilcoxon-Mann-Whitney test.

**Supplementary Table 3**: Quantification and composition of particles > 5 µm observed on the membrane 0.2 µm filters (non lipidic infusion) and 1.2 µm filter (lipids) detected at 100x magnification. Ex-vivo test were designed as for infusions in a 1000g preterm infant during 24 hours (for lipids) and 72 hours (for non lipidic infusion). In-vivo test refers to filters collected in 5 patients of the clinical trial after 24 hours (for lipids) and 72 hours (for non lipids).

**Supplementary Figure 1**: Flow chart of recruited patients.

**
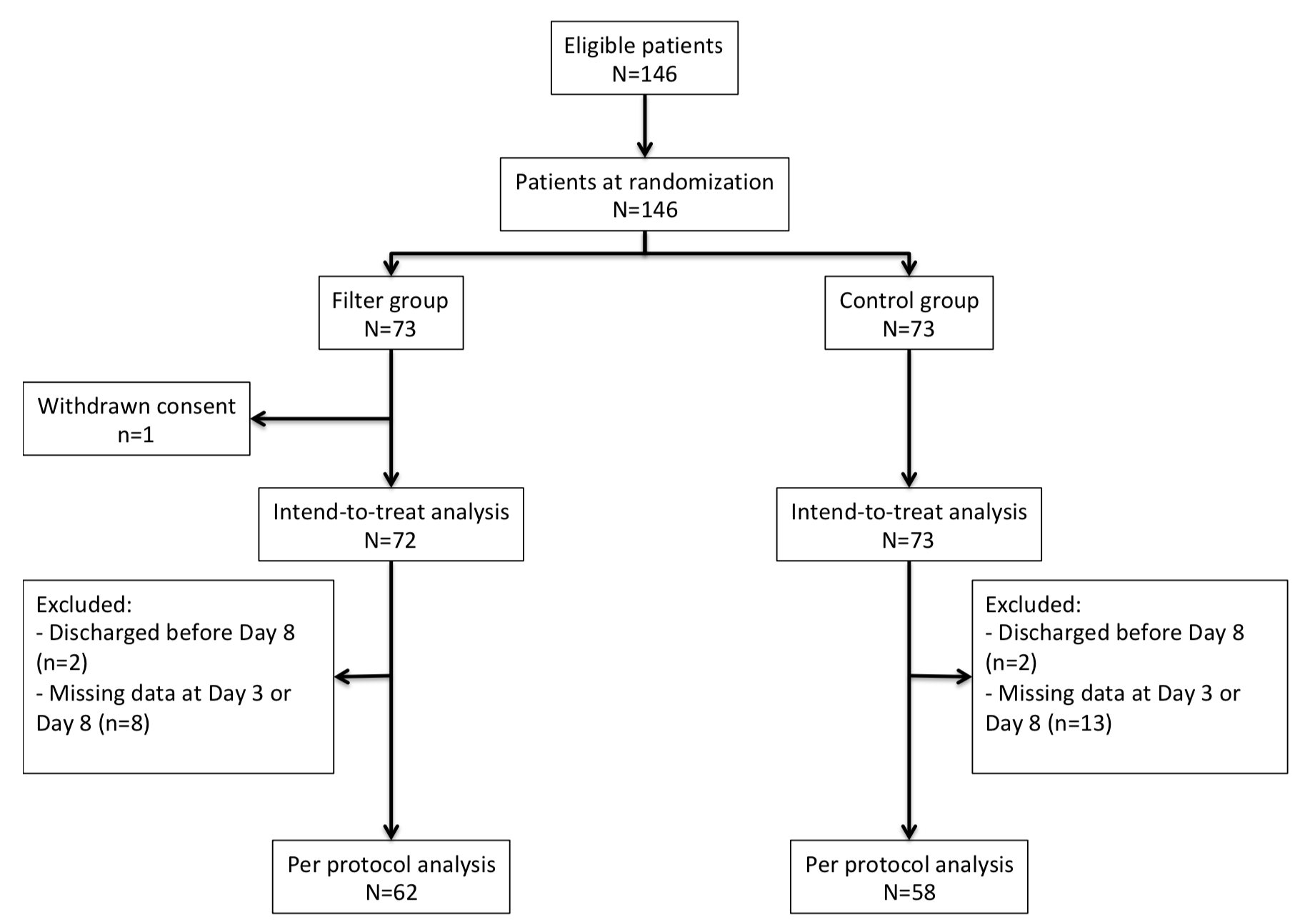
**

**Supplementary Figure 2**: Set-up of *ex vivo* experiments.

**
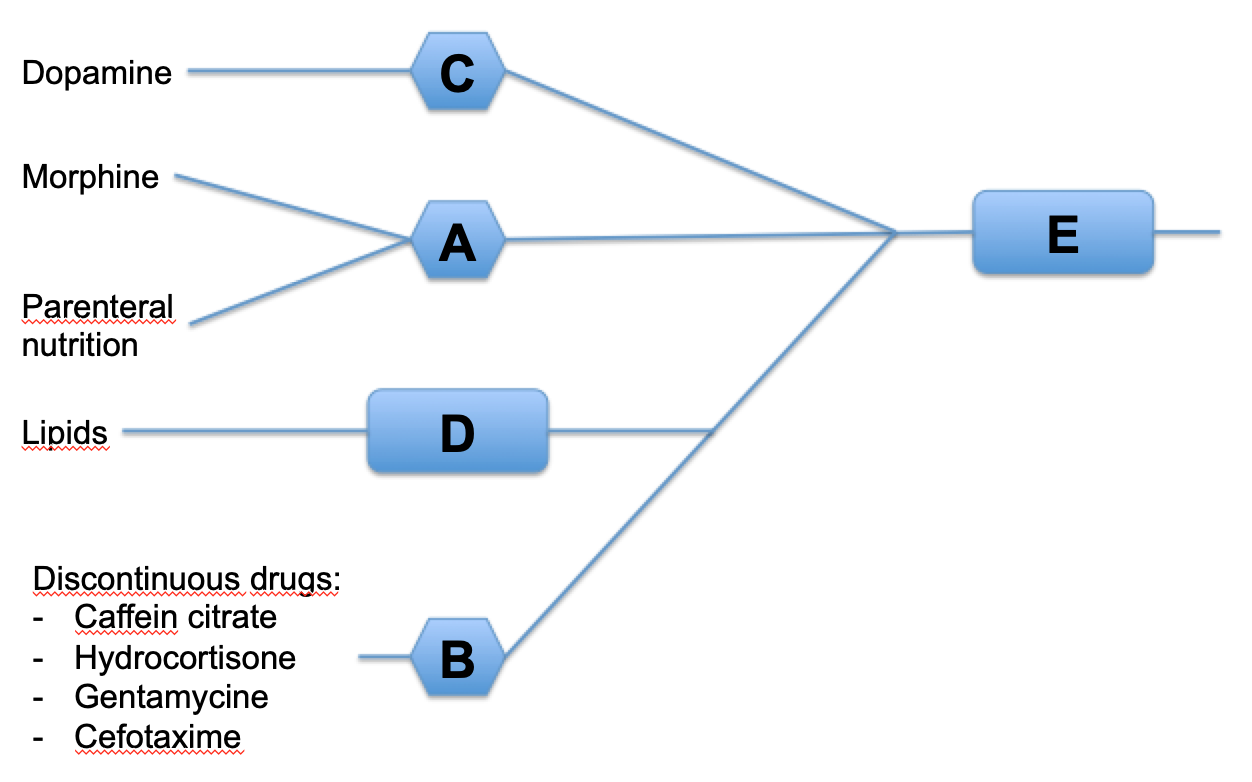
**

**Supplementary Figure 3:** SEM images of particles in the uptream side of membranes from NEO96E filters. Arrows point particles > 5μm. Circles point micro-particles aggregates below level of detection.

**
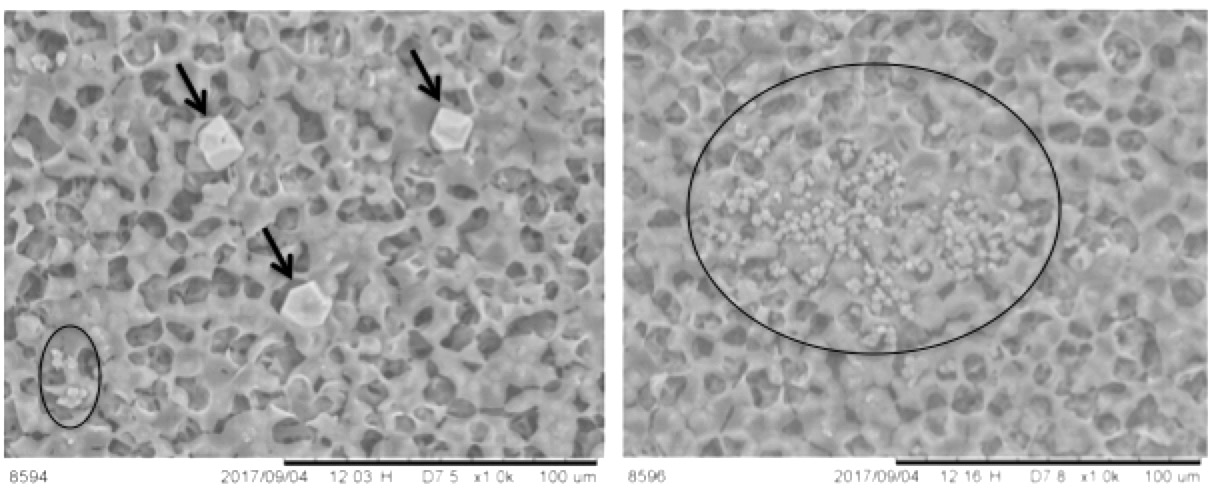
**

**Supplementary Table 1 :** Change in cytokine serum concentration between Day 3 and Day 8 with (ITT) and without (PP) multiple imputations of missing data.

|  | **Cytokine** | **Control group** | **Filter group** | **p-value** |
| --- | --- | --- | --- | --- |
| **ITT** | **Δ IL1β**   Median (IQR)   Min ; Max | N=73  1.01 (-0.14 ; 3.69)  -6.69 ; 7.28 | N=72  0.48 (-0.32 ; 4.01)  -3.19 ; 54.84 | 0.66 |
|  | **Δ IL6**   Median (IQR)   Min ; Max | N=73  -1.25 (-12.99 ; 0.14)  -103.28 ; 49.30 | N=72  -1.11 (-7.50 ; 1.13)  -202.46 ; 37.49 | 0.83 |
|  | **Δ IL8**   Median (IQR)   Min ; Max | N=73  -10.50 (-34.66 ; 3.56)  -347.94 ; 223.35 | N=72  -14.70 (-42.16 ; 5.52)  -1056.40 ; 1284.12 | 0.74 |
|  | **Δ TNFα**   Median (IQR)   Min ; Max | N=73  7.25 (-9.66 ; 20.89)  -93.77 ; 50.24 | N=72  2.99 (-16.12 ; 12.79)  -123.13 ; 191.86 | 0.26 |
| **PP** | **Δ IL1β**   Median (IQR)   Min ; Max | N=36  -0.08 (-0.34 ; 0.60)  -6.69 ; 7.36 | N=42  -0.21 (-0.77 ; 0.30)  -3.19 ; 54.84 | 0.16 |
|  | **Δ IL6**   Median (IQR)   Min ; Max | N=52  -0.85 (-2.93 ; -0.08)  -105.07 ; 49.30 | N=54  -0.93 (-2.85 ; 0.82)  -202.46 ; 37.31 | 0.60 |
|  | **Δ IL8**   Median (IQR)   Min ; Max | N=57  -10.50 (-27.77 ; 0.57)  -239.67 ; 112.80 | N=62  -14.70 (-41.70 ; -0.36)  -1056.40 ; 1284.12 | 0.33 |
|  | **Δ TNFα**   Median (IQR)   Min ; Max | N=56  6.90 (-10.19 ; 20.96)  -93.77 ; 50.24 | N=58  4.76 (-10.92 ; 16.79)  -123.13 ; 191.86 | 0.65 |

**Supplementary Table 2 :** Changes in the serum concentrations (pg/mL) of the panel of 27 cytokines in Filter group compared to Control group between Day 3 and Day 8. Values are expressed as median [interquartile range] and statistical comparisons were performed using Wilcoxon-Mann-Whitney test.

| Cytokine | Control group  (N=73) | Filtered  (N=72) | Missing values | p-value |
| --- | --- | --- | --- | --- |
| IL1β | -0.08 [-0.34 ; 0.60] | -0.21 [-0.77 ; 0.30] | 37/31 | 0.16 |
| IL6 | -0.85 [-2.93 ; -0.08] | -0.93 [-2.85 ; 0.82] | 21/19 | 0.60 |
| IL8 | -10.5 [-27.8 ; 0.6] | -14.7 [-41.7 ; -0.4] | 16/11 | 0.33 |
| TNFα | 6.9 [-10.2 ; 21.0] | 4.8 [-10.9 ; 16.8] | 17/15 | 0.65 |
| IL1ra | -744 [-2710 ; 135] | -674.73 [-4113 ; 195] | 16/11 | 0.95 |
| RANTES | 3148 [177 ; 12702] | 2343 [-2017 ; 7465] | 16/12 | 0.13 |
| IL2 | 0.17 [-0.64 ; 1.03] | 0.69 [-0.71 ; 1.53] | 34/38 | 0.42 |
| IL4 | -0.06 [-0.78 ; 0.23] | -0.04 [-0.45 ; 0.38] | 34/30 | 0.89 |
| IL5 | -3.56 [-9.78 ; 3.59] | -0.99 [-8.13 ; 4.79] | 29/26 | 0.27 |
| IL7 | 0.32 [-1.48 ; 1.87] | 0.70 [-1.73 ; 6.66] | 50/49 | 0.83 |
| IL9 | 4.70 [-12.81 ; 21.99] | -2.34 [-21.97 ; 26.21] | 19/12 | 0.53 |
| IL10 | -2.76 [-5.94 ; -0.57] | -1.14 [-5.47 ; 1.21] | 22/18 | 0.08 |
| IL12 | 0.19 [-0.52 ; 7.26] | -0.43 [-8.53 ; 1.93] | 59/53 | 0.22 |
| IL13 | 0.19 [-0.35 ; 0.81] | -0.14 [-0.88 ; 0.29] | 54/49 | 0.19 |
| IL15 | 36.0 [3.1 ; 60.3] | 36.9 [0.1 ; 78.4] | 53/49 | 0.69 |
| IL17 | -1.43 [-7.62 ; 16.94] | -0.31 [-16.18 ; 14.56] | 32/26 | 0.52 |
| EOTAXIN | -1.91 [-15.83 ; 3.63] | -5.67 [-16.90 ; 4.67] | 16/11 | 0.65 |
| FGF | -2.91 [-18.28 ; 9.14] | -8.16 [-18.49 ; 2.70] | 46/44 | 0.42 |
| GCSF | -41.7 [-97.0 ; 43.6] | -26.0 [-124.0 ; 83.0] | 18/12 | 0.54 |
| GMCSF | 0.24 [-27.89 ; 32.59] | -32.7 [-136.5 ; 0.1] | 54/49 | 0.05 |
| IFNγ | -2.11 [-15.24 ; 10.92] | -0.91 [-25.23 ; 11.32] | 22/20 | 0.86 |
| IP10 | -11.1 [-129.1 ; 64.5] | -41.0 [-154.8 ; 50.3] | 16/11 | 0.46 |
| MCP1 | -33.3 [-162.2 ; 38.4] | -28.3 [-114.2 ; 48.6] | 18/14 | 0.56 |
| MIP1a | -0.53 [-2.22 ; 1.09] | -0.56 [-2.00 ; 1.60] | 17/11 | 0.94 |
| PDGFbb | 51.8 [-20.5 ; 578.2] | 24.2 [-62.2 ; 427.7] | 16/11 | 0.24 |
| MIP1β | 7.70 [-25.08 ; 61.56] | 4.67 [-47.51 ; 49.75] | 16/11 | 0.47 |
| VEGF | 0.99 [-98.18 ; 93.92] | -15.4 [-93.7 ; 89.4] | 40/37 | 0.77 |

**Supplementary Table 3:** Quantification and composition of particles > 5 µm observed on the membrane 0.2 µm filters (non lipidic infusion) and 1.2 µm filter (lipids) detected at 100x magnification. Composition of particles was assessed using EDX spectroscopy. Ex-vivo test were designed as for infusions in a 1000g preterm infant during 24 hours (for lipids) and 72 hours (for non lipidic infusion). In-vivo test refers to filters collected in 5 patients of the clinical trial after 24 hours (for lipids) and 72 hours (for non lipids).

| **Drugs / infusion** | **Filter position** | **N** | **Number of particles >5 µm** | **Composition** |
| --- | --- | --- | --- | --- |
| **Ex-vivo** |  |  |  |  |
| Parenteral + morphine | A | 4 | >100 / 12 / 0 / 4 | C, O, Cu, S, Se |
| Discontinuous drugs : caffeine, hydrocortisone, cefotaxime | B | 4 | 0 / 0 / 0 / 0 | C, O |
| Dopamine | C | 4 | 0 / 0 / 0 / 0 | C, O, Cu, Cl |
| Lipids | D | 2 | 6 / 9 | C, O, S |
| Final filtration | E | 4 | 2 / 6 / 0 / 0 | C, O, Cu |
| **In-vivo** |  |  |  |  |
| Parenteral nutrition alone | A | 5 | 19 / 4 / 0 / 0 / 0 | C, O, Ca |
| Parenteral nutrition + continuous vancomycine + NaCl | A | 1 | 21 | C, O, Ca |
| Caffeine alone | B | 1 | 0 | C, O |
| Caffeine + hydrocortisone + cefotaxime + amikacin | B | 1 | 0 | C, O |
| Micafungin + cefotaxime + vancomycine + caffeine + acetaminophen | B | 1 | 1 | C, O |
| Vancomycine + zidovudine + hydrocortisone + caffeine | B | 1 | 1 | C, F, O |
| Dopamine | C | 1 | 0 | C, O |
| Lipids | D | 5 | 40 / 0 / 0 / 0 / 10 | C, O, S, Fe, Cr |

C: Carbon, O: Oxygen, F: Fluorine, Cu: Copper, S: Sulfur, Se: Selenium, Ca: Calcium, Fe: Iron, Cr: Chrome.
